# Some Valuable Products for the Treatment of Diseases of Bacterial Origin

**Published:** 1912-10

**Authors:** 


Some Valuable Products for the Treatment of Diseases of Bacterial Origin.—Since the advent of diphtheria antitoxin, it is doubtful if any new remedial agent has elicited greater interest than is now being manifested in the bacterial derivatives known as Phylacogens. These products were originated by Dr. A. F. Schafer, of California, the method of preparation and technique of application being first presented to the San Joaquin Medical Society in Fresno. To the uninitiated it may be said that the term Phylacogen (pronounced phy-lac-o-gen) means "phylaxin producer," being derived from two Greek words signifying "a guard" and "to produce." The Phylacogens are sterile aqueous solutions of metabolic substances generated by bacteria grown in artificial media. They are produced from a large variety of pathogenic bacteria, such as the several staphylococci, streptococcus pyogenes, bacillus pyocyaneus, diplococcus pneumoniæ, bacillus typhosus, bacillus coli communis, streptococcus rheumaticus, streptococcus erysipelatis, etc.

Four Phylacogens are now offered to the medical profession: Mixed Infection Phylacogen (used in the treatment of bacterial diseases of unknown etiology), Rheumatism Phylacogen, Erysipelas Phylacogen, and Gonorrhea Phylacogen. They have been thoroughly tested clinically and are said to be producing excellent results in the treatment of the various pathological conditions in which they are indicated. They are administered hypodermically —subcutaneously or intravenously—preferably by the former method, the latter being advised only in cases in which a quick result is demanded. They are supplied in hermetically sealed glass vials of 10 c.c. capacity.

The Phylacogens are prepared and marketed by Parke, Davis & Co., who have recently issued a 24-page pamphlet which describes them in detail—the process of manufacture, therapeutic indications, dosage, methods of administration—everything, in fact, that needs to be known by the man who desires to use Phylacogens. Every physician in general practice, every practitioner who desires to keep abreast of the latest advances in bacterial therapy should have a copy of this valuable booklet. Write to Parke, Davis & Co., at their general offices in Detroit, Mich., ask for the "Phylacogen pamphlet," and mention this JOURNAL.

